# Oncolytic peptides DTT-205 and DTT-304 induce complete regression and protective immune response in experimental murine colorectal cancer

**DOI:** 10.1038/s41598-021-86239-6

**Published:** 2021-03-24

**Authors:** Karianne Giller Fleten, J. Johannes Eksteen, Brynjar Mauseth, Ketil André Camilio, Terje Vasskog, Baldur Sveinbjørnsson, Øystein Rekdal, Gunhild M. Mælandsmo, Kjersti Flatmark

**Affiliations:** 1 Department of Tumor Biology, Institute for Cancer Research, The Norwegian Radium Hospital, Oslo University Hospital, Montebello, 0379 Oslo, Norway; 2 Faculty of Medicine, Institute of Clinical Medicine, University of Oslo, Oslo, Norway; 3 NORCE Norwegian Research Centre AS, SIVA Innovation Centre, Tromsø, Norway; 4 Lytix Biopharma, Oslo, Norway; 5 Department of Pharmacy, The Arctic University of Norway – University of Tromsø, Tromsø, Norway; 6 Institute of Medical Biology, Faculty of Health Sciences, The Arctic University of Norway – University of Tromsø, Tromsø, Norway; 7 Department of Gastroenterological Surgery, The Norwegian Radium Hospital, Oslo University Hospital, Oslo, Norway

**Keywords:** Gastrointestinal cancer, Immunotherapy

## Abstract

Oncolytic peptides represent a novel, promising cancer treatment strategy with activity in a broad spectrum of cancer entities, including colorectal cancer (CRC). Cancer cells are killed by immunogenic cell death, causing long-lasting anticancer immune responses, a feature of particular interest in non-immunogenic CRC. Oncolytic peptides DTT-205 and DTT-304 were administered by intratumoral injection in subcutaneous tumors established from murine CRC cell lines CT26 and MC38, and complete regression was obtained in the majority of animals. When cured animals were rechallenged by splenic injection of tumor cells, 1/23 animals developed liver metastases, compared to 19/22 naïve animals. Treatment with both peptides was well tolerated, but monitoring post-injection hemodynamic parameters in rats, less extensive changes were observed with DTT-205 than DTT-304, favoring DTT-205 for future drug development. DTT-205 was subsequently shown to have strong in vitro activity in a panel of 33 cancer cell lines. In conclusion, both peptides exerted a strong inhibitory effect in two immunocompetent CRC models and induced a systemic effect preventing development of liver metastases upon splenic rechallenge. If a similar effect could be obtained in humans, these drugs would be of particular interest for combinatory treatment with immune checkpoint inhibitors in metastatic CRC.

## Introduction

Metastatic colorectal cancer (mCRC) is a leading cause of cancer related mortality worldwide, with the liver as the most common metastatic site^1^. Surgery is a curative treatment option in a subgroup of mCRC patients with limited disease burden, while the majority of cases will receive palliative systemic chemotherapy only^2,3^. Recently, immunotherapy, including inhibitors of programmed death protein 1 (PD-1) and its ligand PD-L1, has been shown to increase the survival in patients with different types of cancer, including melanoma, lung cancer, and renal cell cancer^4,5^. However, with the exception of the small subgroup of microsatellite instable (MSI) tumors, CRC remains a non-immunogenic cancer that responds poorly to immunotherapy^4,6^. Extensive research efforts are therefore being mounted to develop strategies for inducing immune responses in non-immunogenic cancers. Clinical development of oncolytic therapies is gaining momentum, with one drug i.e. T-VEC already approved for treating patients. A promising new approach to oncolytic therapy is the use of oncolytic peptides. These peptides are based on host defense peptides (cationic antimicrobial peptides; CAPs) which are part of the innate immune system of virtually all living organisms, protecting the organisms against foreign pathogens^7^. CAPs preferentially kill cancer cells over non-cancerous cells, due to higher expression of anionic macromolecules on the cell membranes, and kill dividing and non-dividing cells as well as chemotherapy resistant cells by their membranolytic action^7–9^. Following intralesional injection, tumor cells are rapidly killed by immunogenic cell death with release of damage associated molecular patterns (DAMPs) and tumor antigens. This will result in recruitment and activation of dendritic cells with subsequent T-cell activation and long-term tumor-specific immune response^10^. Several CAPs have shown to cure cancer in vivo and also to induce a long-lasting immune response^11–14^. Some, such as LTX-315 has even progressed to early phase clinical trials (NCT01058616, NCT01223209, NCT01986426, NCT03725605).

Oncolytic peptides offer several potential advantages over oncolytic viruses in terms of efficacy, safety regulations, manufacturing and handling. But more importantly, oncolytic peptides are less likely to have unwanted immune effects. For example, unlike viruses, peptides cannot self-replicate and they are generally too small to induce an adverse immune response against itself. Furthermore, proteolytic degradation into harmless metabolites will usually prevent systemic accumulation and toxicity as is often observed during conventional chemotherapy^15,16^. Intralesional oncolytic peptide therapy therefore represents a novel local treatment modality with systemic effect and, potentially, an improved therapeutic window.

In the present study, the therapeutic efficacy of administering local injections of DTT-205 and DTT-304 was investigated in subcutaneous (s.c.) tumors in two syngeneic models of murine CRC. Cured animals were rechallenged by splenic injection of tumor cells for establishment of liver metastases. In vitro pharmacological and in vivo cardiovascular safety studies were subsequently performed as a preliminary assessment of the peptides’ therapeutic potential. Finally, an extended cytotoxicity profile for DTT-205 was determined in a large panel screen with additional cancer cell lines in order to pave the way for in vivo studies in other cancer models.

## Results

### Peptide injections induced complete regression of CT26 and MC38 tumors

**Figure 1:**
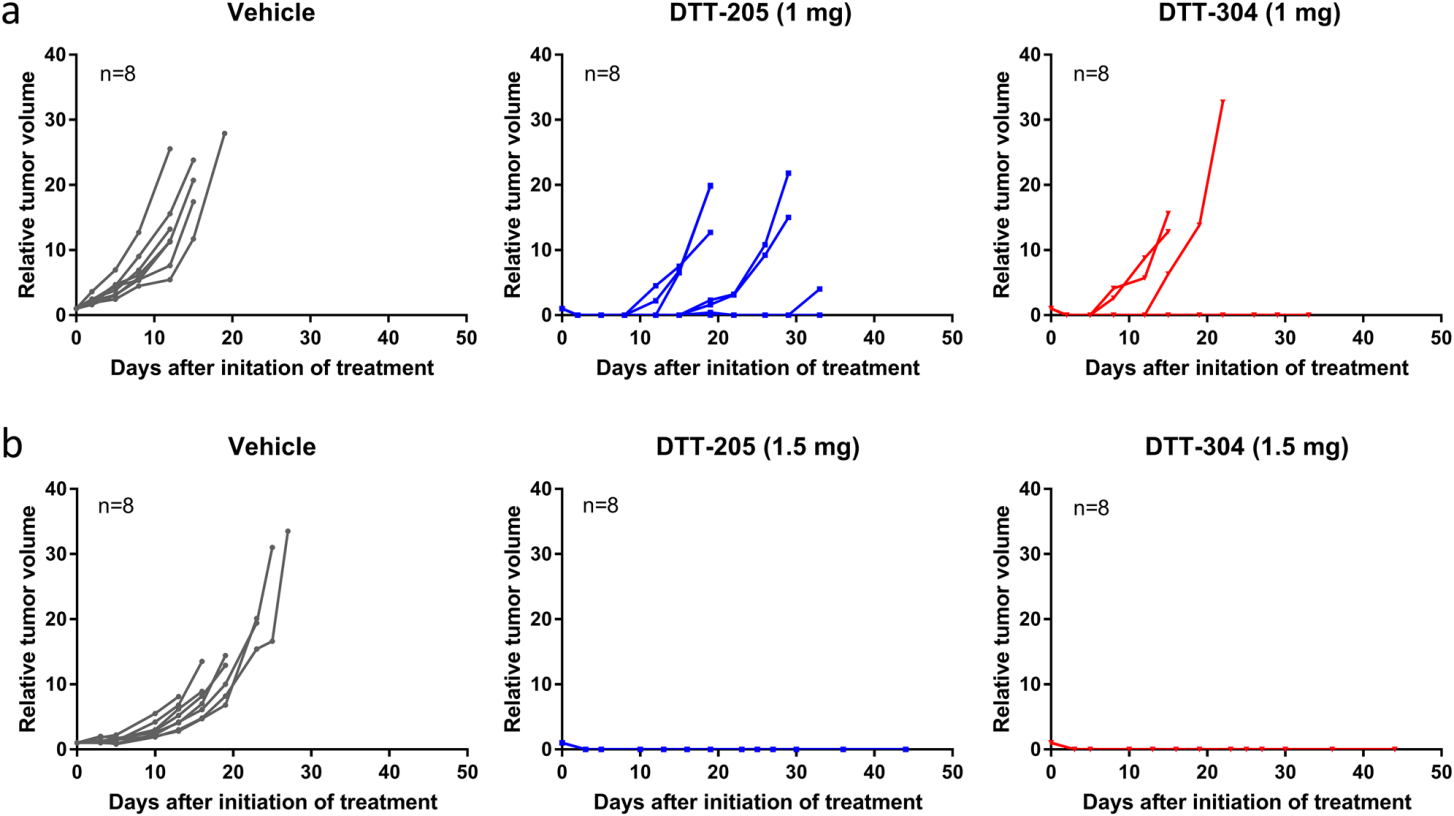
Response to DTT-205 or DTT-304 treatment in CT26 tumors. (**a**) Tumor growth curves of CT26 tumors treated with vehicle, 1 mg DTT-205 or 1 mg DTT-304. Tumors were treated for three consecutive days. (**b**) Tumor growth curves of CT26 tumors treated with vehicle, 1.5 mg DTT-205 or 1.5 mg DTT-304 for four consecutive days.

CT26 tumors were established in Balb/c mice, and two intratumoral injection regimens were investigated. In the first experiment, injections of vehicle, DTT-205 (1 mg) or DTT-304 (1 mg) were administered for three consecutive days (8 tumors in each group). Tumors treated with vehicle grew rapidly, and the mice were sacrificed because of large tumor size (> 15 mm) median 14 days after treatment initiation. All tumors treated with peptide initially disappeared, and complete regression was observed in 2 and 5 animals treated with DTT-205 and DTT-304, respectively, while regrowth occurred in 6 of 8 tumors treated with DTT-205 and 3 of 8 tumors treated with DTT-304 (Fig. 1a). In a second experiment, to investigate if administration of a higher peptide dose would increase the cure rate, injections of 1.5 mg were given for four consecutive days. Again, vehicle-treated tumors grew rapidly, and the mice were sacrificed median 19 days after treatment initiation, while all peptide-treated tumors regressed completely and no regrowth was observed (Fig. 1b).

**Figure 2:**
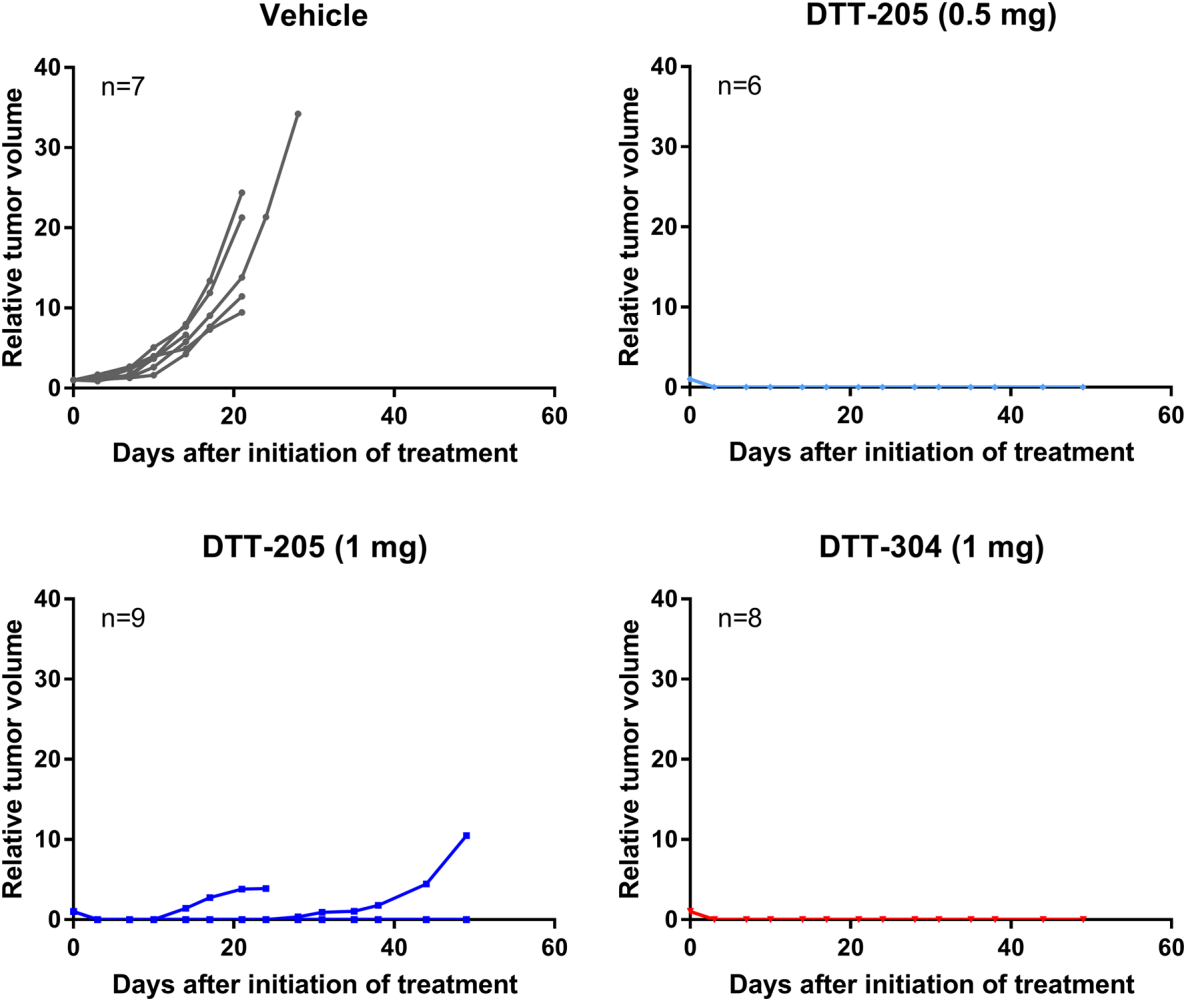
Response to DTT-205 or DTT-304 treatment in MC38 tumors. Tumor growth curves of MC38 tumors treated with saline, 0.5 mg DTT-205, 1 mg DTT-205 or 1 mg DTT-304. Mice were treated for three consecutive days.

In the MC38 model, s.c. tumors were established in C57Bl/6 mice and treated with injections of vehicle (n = 7), DTT-205 (0.5 mg; n = 6), DTT-205 (1 mg; n = 9), or DTT304 (1 mg; n = 8) for three consecutive days. Tumors treated with vehicle grew rapidly and the mice were sacrificed within 28 days of treatment initiation. Complete regression was observed in 21 of 23 peptide-treated animals, while two tumors treated with 1 mg DTT-205 regrew after 14 and 28 days (Fig. 2).

### Curative peptide treatment prevented development of liver metastases

To investigate if curative treatment with DTT-205 or DTT-304 would protect animals from developing liver metastases, mice cured for s.c. tumors by peptide treatment were rechallenged by splenic injection of CT26 or MC38 cells in Balb/c or C57Bl/6 mice, respectively. Naïve mice injected with the same number of cells served as experimental controls.

**Figure 3:**
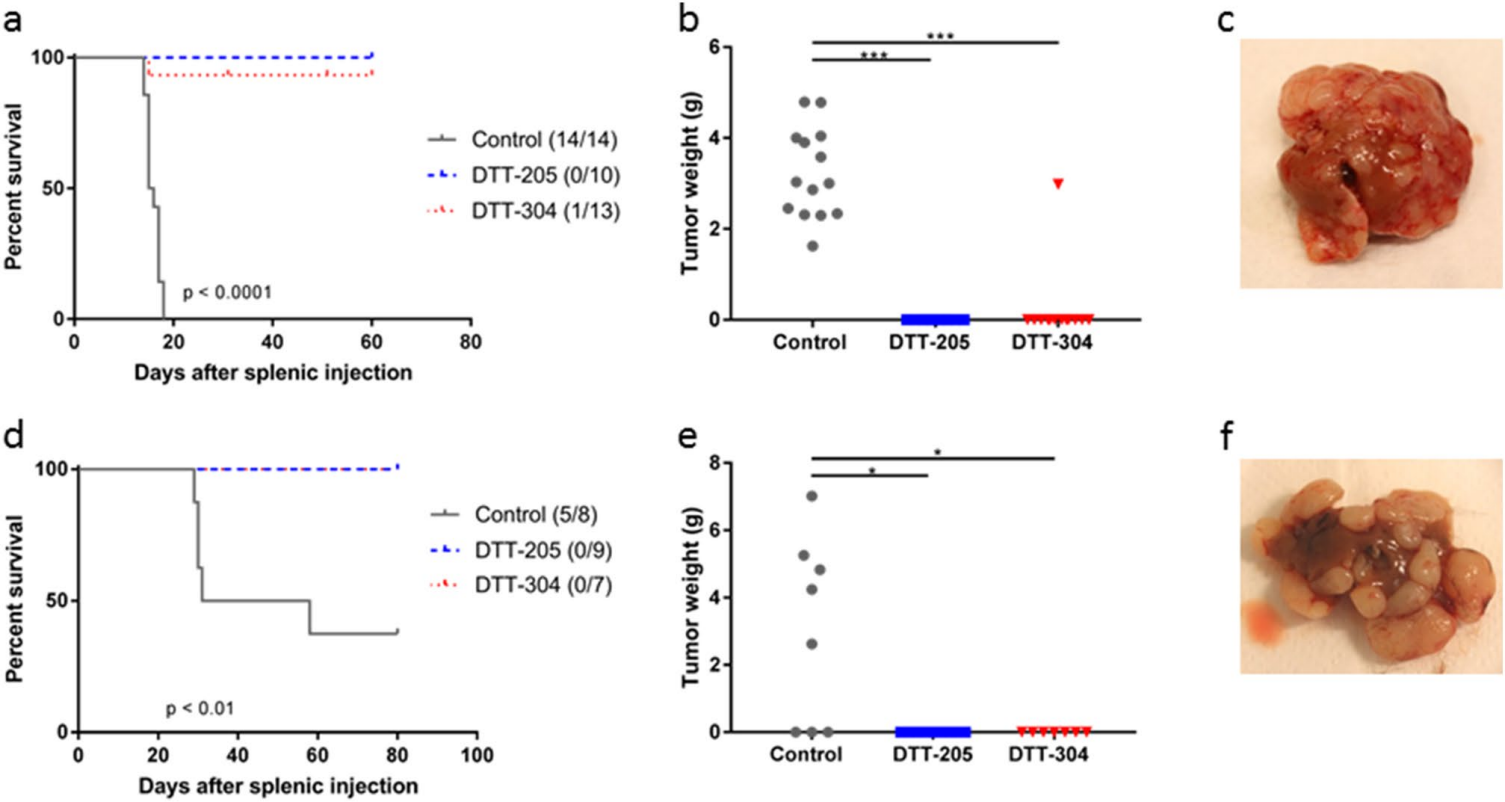
Survival after rechallenge with splenic injection of tumor cells. (**a**) Kaplan–Meier curves comparing naïve and previously cured Balb/c mice after splenic injection of CT26 cells. In parentheses, the number of animals that developed liver metastases/total number of mice. (**b**) Tumor weight in the liver in Balb/c mice after splenic injection (tumor was dissected from liver tissue). (**c**) Representative image of CT26 tumor in the liver in Balb/c mice. (**d**) Kaplan–Meier curves comparing naïve and previously cured C57Bl/6 mice after splenic injection of MC38 cells. In parentheses, the number of animals that developed liver metastases/total number of mice. (**e**) Tumor weight in the liver in C57Bl/6 mice after splenic injection. (**f**) Representative image of MC38 tumor in the liver in C57Bl/6 mice. *p < 0.05, **p < 0.01, ***p < 0.001.

All Balb/c control animals had to be sacrificed within 20 days of splenic injection due to tumor growth, and liver tumors (3.26 ± 1.02 g) and bloody ascites (1.21 ± 0.52 g) were detected at autopsy. None of the 10 mice previously cured by DTT-205 and only 1 of 13 mice previously cured by DTT-304 developed liver metastases. The previously cured mouse that developed liver metastases had similar survival (15 days) and tumor burden (2.98 g tumor and 1.4 mL ascites) as the control mice. Two of the mice cured by DTT-304 had to be sacrificed after 31 and 51 days because of ulcerations at the previous s.c. treatment site (Fig. 3a,b).

None of the 16 C57Bl/6 mice previously cured by peptide injections developed liver tumors, while 5 of 8 naïve mice developed tumor (Fig. 3d). C57Bl/6 mice that developed tumor had 4.97 ± 1.77 g tumor in the liver at time of sacrifice (Fig. 3e). Ascites was present in one mouse (0.4 g). Representative images of tumor-bearing livers are shown in Fig. 3c,f.

### Induction of necrosis by peptide treatment

**Figure 4:**
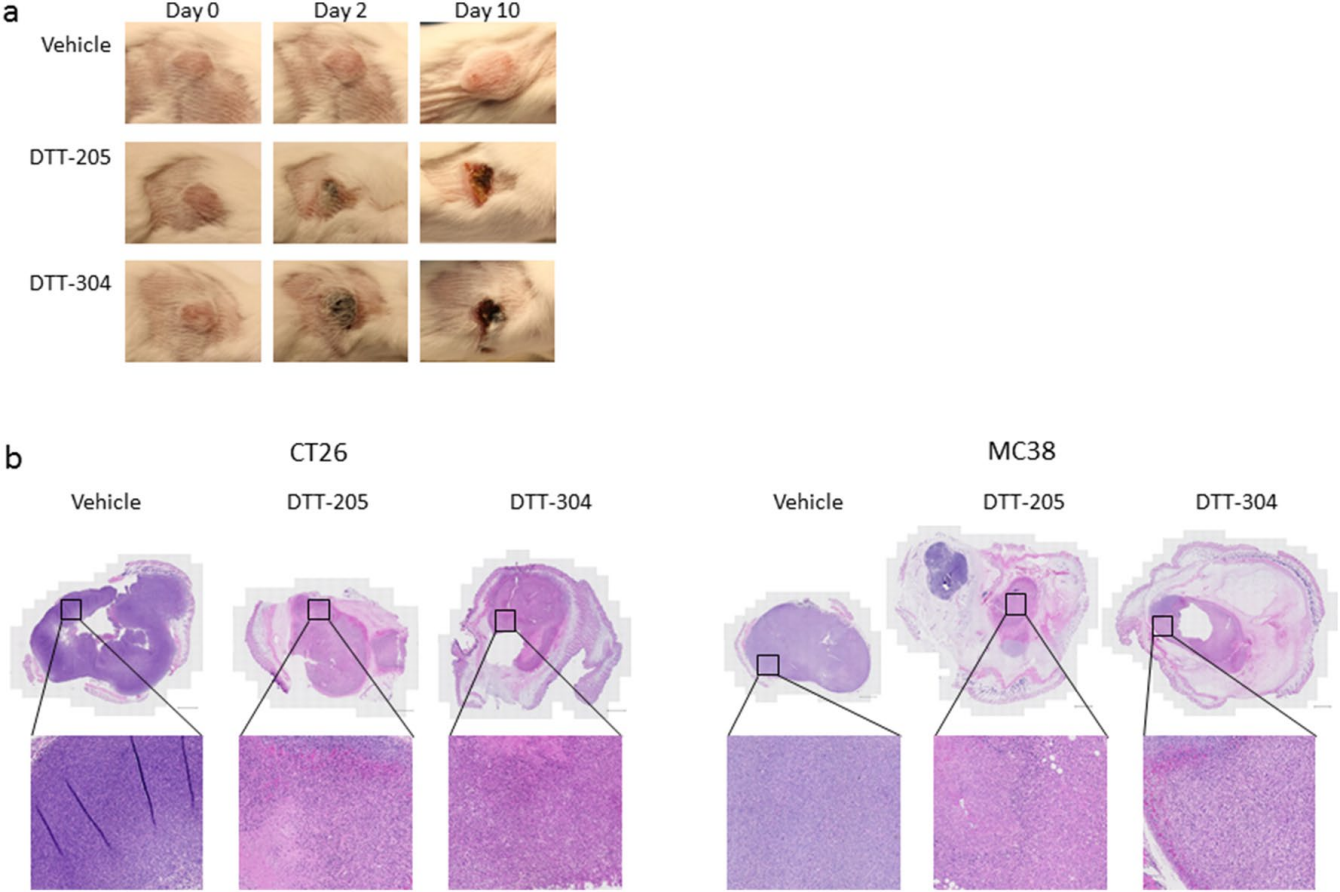
Macroscopic and histological analyses of CT26 and MC38 tumors after treatment with DTT-205 and DTT-304. (**a**) Images of CT26 tumors before treatment, and at day 2 and 10 after three injections with vehicle, 1 mg DTT-205 or 1 mg DTT-304. (**b**) HE staining of whole tumor (scale bar 1000 µm) and enlargement of selected areas of CT26 and MC38 treated with 2 injections of 1 mg DTT-205, DTT-304 or saline. Tumors were harvested 48 h after the first peptide injection.

Intratumoral injection with peptide induced rapid swelling of the tissue surrounding the s.c. tumors, interpreted as local inflammation. Images taken before, and at various time points after treatment showed growth of the tumor and development of necrosis in Balb/c mice (Fig. 4a). No macroscopic differences were observed between tumors treated with DTT-205 or DTT-304. In a separate experiment, tumors were treated with two daily injections (1 mg) of DTT-205 or DTT-304 and harvested for histological analyses 48 and 120 h after the first injection (n = 4). Treatment with DTT-205 and DTT-304 induced necrosis and significant reduction of tumor size resulting in no to little viable tumor tissue in CT26 tumors harvested after 48 and 120 h and in MC38 tumors harvested after 48 h (Fig. 4b). In MC38 tumors harvested after 120 h, there was little viable tumor tissue after treatment with DTT-304 while a considerable amount of viable tumor tissue was left in 3 of 4 tumors treated with DTT-205.

### Rapid proteolytic degradation

**Figure 5:**
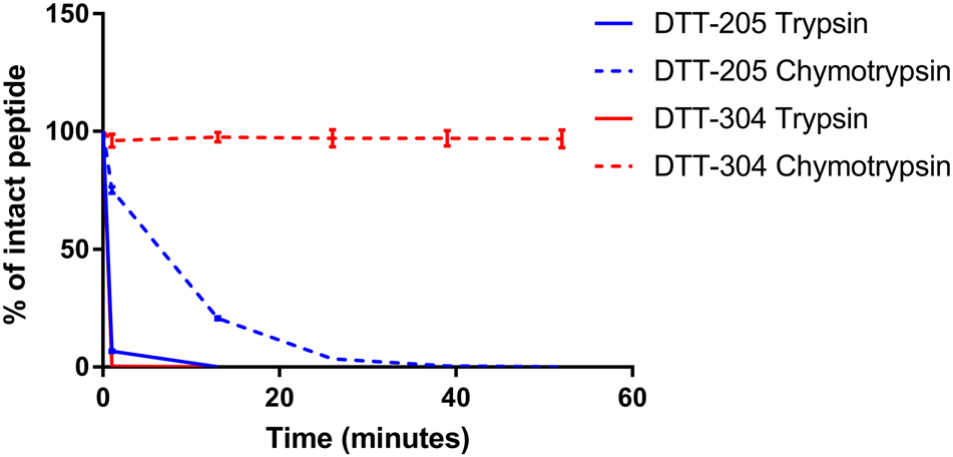
Degradation of peptides by trypsin or chymotrypsin. Degradation of DTT-205 and DTT-304 after administration of trypsin or chymotrypsin every 13 min, shown as % of remaining peptide. Error bars indicate standard deviation.

It is expected that oncolytic peptides, after exerting its desired therapeutic effect, will be degraded and its metabolites rapidly removed from circulation. As a result, oncolytic peptides, unlike chemotherapeutics, are not expected to accumulate in the patient’s body, and this represents a major therapeutic advantage. The degradation assay performed in this study, illustrated that as expected, both DTT-205 and DTT-304 were rapidly degraded in vitro by enzymes having cleavage sites in the peptide sequences (Fig. 5). DTT-304 does not have a cleavage site for chymotrypsin and consequently no degradation was observed at the end of the experiment. MS analysis indicated that DTT-205 were broken down into several species of short peptide fragments of which the majority were 2–8 amino acids in length.

### Assessment of adverse effects of DTT-205 and DTT-304

**Figure 6:**
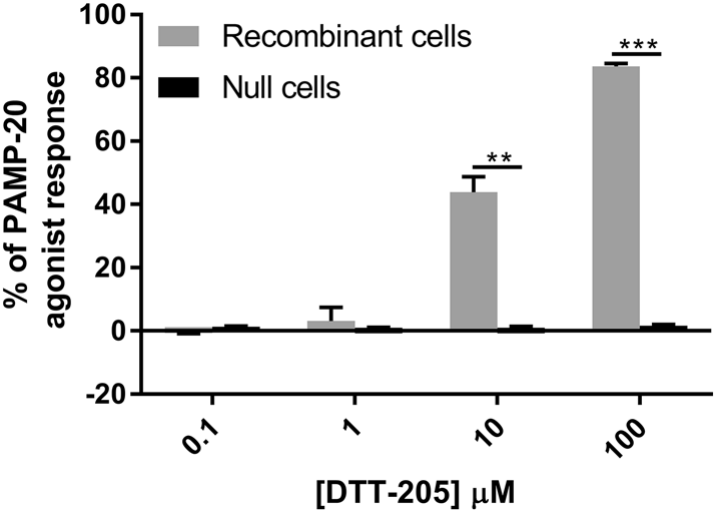
Calcium flux after treatment with DTT-205. Release of calcium flux, shown as % of calcium flux induced by PAMP-20 agonist in recombinant and null cells after treatment with increasing doses of DTT-205. Error bars indicate standard deviation. **p < 0.01, ***p < 0.001.

It is anticipated that following intratumoral injection, some of the peptide will escape into the blood stream, which could cause systemic adverse effects, typically hypotension. A possible mechanism for this could be binding of DTT-205 to the MRGX2 receptor. A dose dependent increase in calcium flux indicating binding to the receptor was observed in recombinant cells when treated with increasing concentration of DTT-205, indicating that DTT-205 act as a MRGX2 receptor agonist (Fig. 6). No effect was observed in the host cells without MRGX2 expressed (null cells).

**Figure 7:**
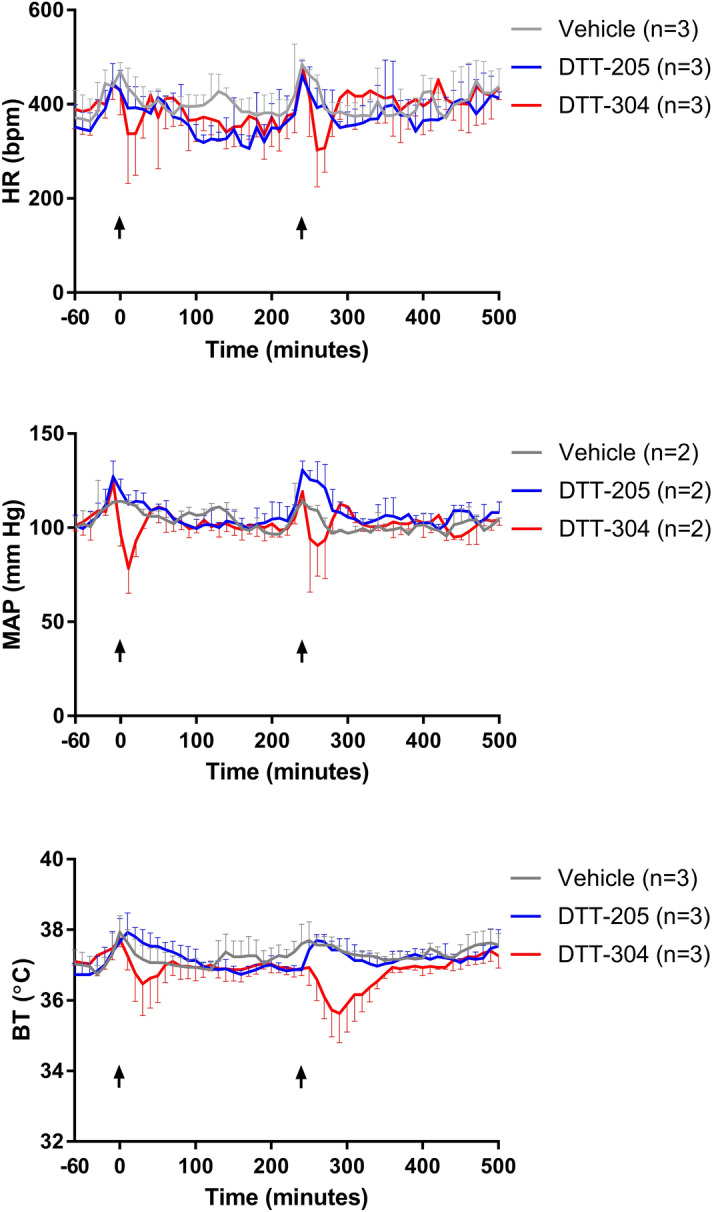
Cardiovascular and temperature assessment in rats after treatment with DTT-205 or DTT-304. Heart rate (HR), mean arterial pressure (MAP) and body temperature (BT) in rats treated with DTT-205 or DTT-304 in the period − 60 to 500 min after injection. Arrows indicate time of injection. Error bars indicate standard deviation.

To further explore the cardiovascular activity of the peptides, DTT-205 and DTT-304 were injected intravenously (i.v) in rats, and body temperature, heart rate, mean arterial pressure (Fig. 7), systolic arterial pressure, diastolic arterial pressure, and pulse pressure (Supplementary Fig. S1) were measured. DTT-205 had no effect on cardiovascular parameters or body temperature, while DTT-304 induced a significant decrease in mean arterial pressure after the first dose (p = 0.01) and in body temperature after the second dose (p = 0.02) compared to vehicle treated rats. For the other parameters, a trend towards reduction was observed for DTT-304, but the differences were not significant. Only measurements from 60 min before to 500 min after the first injection are shown in Fig. 7. The full graph showing measurement from 60 min before to 1440 min after the first injection is shown in Supplementary Fig. S2. Based on the results from the preliminary safety studies, DTT-205 was judged to be superior to DTT-304 in terms of therapeutic index and was therefore selected as lead compound.

### DTT-205 is cytotoxic against a variety of cancer cell lines

The cell viability assay revealed that DTT-205 is highly cytotoxic against most types of cancer cells, with IC_50_ values in the range of 1.5–6 µM for the majority of the cell lines. The only cancer type that was not as responsive to the peptide was kidney cancer with IC_50_ values of 8.3–20.9 µM. DTT-205 also had some toxicity towards the normal cell types that were tested with IC_50_ values varying from 1.8 to 12.9 µM (Table 1).

**Table 1:** IC_50_ values of DTT-205 against various cell lines.

**#**	Cell line	Disease/histological subtype	Origin	IC_50_ (µM)
1	CCD-18Co	NORMAL	Colon	4.4
2	MRC-5	NORMAL, fœtal	Fibroblast	8.8
3	BEAS-2B	NORMAL, broncheal, virus tranformed	Lung	1.8
4	HMVEC	NORMAL, microvascular endothelial	Lung	12.9
5	UM-UC-3	Transitional papilloma	Bladder	4.2
6	RT-4	Transitional cell carcinoma	Bladder	6.1
7	LN-229	Glioma	Brain	5.1
8	U-87 MG	Glioblastoma	Brain	4.5
9	BT-474	Invasive ductal carcinoma, primary	Breast	3.0
10	MCF-7	Invasive ductal carcinoma, pleural effusion metastasis	Breast	3.7
11	MDA-MB-231	Invasive ductal carcinoma, pleural effusion metastasis	Breast	3.7
12	HT-29	Colorectal adenocarcinoma	Colon	5.5
13	NCI-H747	Adenocarcinoma	Colon	3.8
14	SW-480	Colorectal adenocarcinoma	Colon	3.2
15	FaDu	Pharynx carcinoma, squamous	Head & neck	3.9
16	KB	Mouth epidermoid carcinoma	Head & neck	3.2
17	786-O	Clear cell adenocarcinoma	Kidney	8.3
18	A-498	Carcinoma	Kidney	20.9
19	Caki-1	Clear cell carcinoma, skin metastasis	Kidney	10.9
20	Hep G2	Hepatocellular carcinoma	Liver	3.8
21	SK-HEP-1	Adenocarcinoma, ascites metastasis	Liver	5.1
22	Calu-3	Adenocarcinoma; non-small cell lung cancer	Lung	4.1
23	NCI-H1975	Adenocarcinoma; non-small cell lung cancer	Lung	5.9
24	NCI-H146	Carcinoma; small cell lung cancer carcinoma	Lung	3.4
25	NCI-H209	Carcinoma; small cell lung cancer carcinoma	Lung	1.5
26	A2780	Adenocarcinoma	Ovary	3.1
27	NIH:OVCAR-3	Adenocarcinoma, ascites metastasis	Ovary	8.9
28	SW-626	Adenocarcinoma	Ovary	4.2
29	BxPC-3	Adenocarcinoma	Pancreas	6.3
30	Capan-1	Adenocarcinoma, liver metastasis	Pancreas	5.5
31	Capan-2	Adenocarcinoma	Pancreas	3.4
32	DU 145	Carcinoma, brain metastasis	Prostate	2.4
33	MDA PCa 2b	Adenocarcinoma, bone metastasis	Prostate	3.1
34	PC-3	Adenocarcinoma, bone metastasis	Prostate	5.1
35	A-375	Malignant melanoma	Skin	1.7
36	A-431	Epidermoid carcinoma	Skin	7.2
37	SK-MEL-28	Malignant melanoma	Skin	1.9

## Discussion

The main finding of this experimental study was that in two syngeneic CRC models, local injections of oncolytic peptides DTT-205 and DTT-304 resulted in complete s.c. tumor regression and cure in 44 of 55 treated animals. Furthermore, when the cured mice were rechallenged with splenic injection of tumor cells, only one of 39 animals developed liver metastases, suggesting that the initial treatment response prevented establishment of liver metastases.

The first generation of DTT peptides was originally designed for treating lymphoma, and to generate peptides with a broader anti-cancer spectrum, DTT-205 and DTT-304 were developed^17^. The two peptides have similar, but not identical, mode-of-actions involving lysosomal localization. In addition, DTT-205 and DTT-304 may influence pro-oxidant and BAX/BAK dependent processes, as inhibition of these also led to reduced efficacy^18^. Intratumoral injections of DTT-205 and DTT-304 have previously been investigated in s.c. models in C57Bl/6 mice using the mouse fibrosarcoma cell line MCA205 and the lung carcinoma cell line TC-1. Both peptides induced regression, indicating that the effect was independent of cancer type. Prior depletion of T-cells using antibodies targeting CD4 and CD8 abolished the sustained growth inhibitory effect. In cured animals, tumors could not be reestablished when rechallenged by s.c. injection^18^. To evaluate the presence of a sustained growth inhibitory effect in our models, mice cured of s.c. tumors were rechallenged using a liver colonization model^19^. The majority of the mice previously cured by peptide treatment (38/39 animals) did not develop liver metastases, which is in accordance with previous findings in other cancer types and tumor locations^13^. Prevention of tumor growth in a secondary site in these experiments further strengthens the hypothesis that oncolytic peptide treatment can induce a systemic long-term immune protective effect. A possible treatment strategy for patients could be to inject the peptides in the primary tumor before removal to possibly prevent establishment of metastases at a later time point.

Previous results have shown that DTT-205 and DTT-304 can trigger immunogenic cell death (ICD) with the release of damage associated molecular patterns such as ATP, HMGB1 and CALR1^18^. Induction of ICD can lead to increased T-cell infiltration in tumors^20,21^, which has been shown to be associated with improved efficacy of immune checkpoint inhibitors^22^ and strategies to increase the infiltration of immune cells in non-immunogenic or cold tumors are of great interest. In mCRC, checkpoint inhibitors have exhibited poor activity in patients with microsatellite stable (MSS) tumors, and this group is therefore a particular focus in ongoing research in the field^23^. It has previously been shown that oncolytic peptide treatment lead to increased tumor infiltration of T-cells^14,24^. In our studies, no increase of T-cell infiltration was observed after treatment, but a possible explanation may be that treated tumors largely consisted of necrotic tissue, making comparisons with vehicle-treated tumors difficult (Supplementary Fig. S3). With the CT26 and MC38 being murine equivalents of MSS and MSI CRC, respectively^25,26^, the subsequent prevention of liver metastasis establishment still suggests that results have potential relevance both in MSS and MSI CRC.

In our experiments, the toxicity of DTT-205 and DTT-304 was low and comparable, with survival rates close to 100%, while a higher toxicity was previously observed in vivo with DTT-304 compared to DTT-205 with survival rates of 72% and 90%, respectively^27^. The underlying cause of toxicity has not been established, but hypotension has been the prime suspect. Since oncolytic peptide therapy involves transdermal injection of peptide directly into the tumor, there is a possibility that some of the injected peptide may escape the injection site to cause systemic side effects. Although DTT-205 was shown to interact with the promiscuous MRGX2 receptor, known to induce histamine release from mast cells and cause hypotension^28^, no major toxicity and no hypotension were observed upon DTT-205 administration in vivo. A possible explanation could be that the peptides are rapidly degraded by proteolytic enzymes (i.e. trypsin and chymotrypsin) present in blood. On the other hand, it might be necessary to investigate potential systemic effects of higher DTT-205 doses, since the DTT-205 dose administered i.v. to rats in the cardiovascular studies (0.3–1 mg/kg) was lower than doses used for tumor injection in mice (25–75 mg/kg).

The extensive cell line screening showed that DTT-205 exhibited high in vitro activity against a broad range of cancer cell types, but also that some normal cells may be affected. These results suggest that DTT-205 has the potential to treat most kinds of tumors, provided that some degree of damage to normal tissue surrounding the tumor is clinically acceptable. Therefore, while DTT-205 has a much broader anti-tumor activity than the more tumor specific peptide predecessors, it also seems to be less selective. Since oncolytic peptides are best suited for tumors that are accessible to local injections, this aspect should be taken into account in the clinical development of DTT-205 as an anti-cancer drug.

In conclusion, oncolytic peptides DTT-205 and DTT-304 both displayed strong growth inhibitory effects in two immunocompetent murine CRC models and induced a long-lasting systemic effect preventing tumor growth in the liver. The observed results are consistent with the peptides inducing an immunogenic response, which could be of particular interest in MSS mCRC for a potential clinical application in combination with immune check point inhibitors or adoptive cell therapies.

## Materials and methods

### Peptide synthesis

DTT-205 and DTT-304 were synthesized with a Prelude instrument (Protein Technologies Inc. Tucson, AZ, USA) using a solid-phase Fmoc-approach. All synthesized peptides were prepared as C-terminal amides. The resin (Rink amide) and Fmoc-amino acids used were standard derivatives purchased from Novabiochem (Merck Millipore, Billerica, MA, USA). Fmoc-derivative activations were done in dimethylformamide (DMF) using HCTU (2-(6-chloro-1H-benzotriazole-1-yl) − 1,1,3,3-tetramethylaminium hexafluorophosphate) and diisopropylethylamine (5 eq and 10 eq relative to the resin, respectively). To ensure complete reactions, double couplings (2 × 30 min) were performed. Coupling reactions were follow-up with a washing (DMF, 3 × 30 s) and Fmoc-deprotection step (20% piperidine in DMF, 5 + 10 min). Following the final Fmoc-deprotection, the completed peptides were cleaved from the resin using a cocktail containing 95% trifluoroacetic acid (TFA), 2.5% MilliQ water (MQ) and 2.5% triisopropylsilane for 3 h. The spent resin particles were removed by passing the peptide-containing cleavage cocktail through a sintered glass funnel. The TFA was removed under reduced pressure (Hei-VAP Advantage rotavapor, Heidolph Instruments, Schwabach, Germany). The fully deprotected peptides were precipitated using cold diethyl ether, afterwards the ether was decanted and the precipitated crude peptide air dried.

### Peptide purification and characterization

MQ water (solvent A) and acetonitrile (MeCN) (solvent B), both modified with 0.1% TFA, were used for analytical and preparative liquid chromatography. Purification was done using an AutoPurification System (Waters, Milford, MA, USA) fitted with a XSelect CSH C18 OBD column (19 × 250 mm, 5 µm). A default gradient of 10–40% solvent B over 30 min with a flow rate of 20 mL/min was used, but was adjusted when necessary. Fractions containing peptides were automatically collected and further analyzed for desired material. Purity analyses were done using an ACQUITY UPLC H-class system fitted with an ACQUITY BEH C18 UPLC column (Waters, 2.1 × 50 mm, 1.7 µm). Detection was done at 200–500 nm using a Waters ACQUITY photodiode array detector. A gradient of 0–50% solvent B over 30 min with a flow rate of 1 mL/min was used. Fractions with a purity of > 95% were pooled and lyophilized (Labconco FreeZone 4.5 Plus, Kansas City, MO, USA) as TFA-salts. The correct molecular weight of the peptides was confirmed on a Waters Xevo G2 Q-TOF with ACQUITY UPLC I-Class system.

### Converting peptides into acetate salts

In order to convert the peptides into acetate salts, the TFA counterions first had to be removed. This was done by dissolving the TFA-containing peptides in MQ and passing it through an Agilent StratoSphere ML-HCO3 MP cartridge (Agilent Technologies, Santa Clara, CA, USA). The free base peptides eluting from the cartridge were collected, a small amount (0.1% v/v) of acetic acid added, and the solution lyophilized. The resulting peptide acetate salt was stored at − 20 °C until further use.

### Cell lines

Two murine CRC cell lines were used for treatment experiments. The CT26 cell line (ATCC, Manassas, VA, USA) was cultured in RPMI-1640 supplemented with 10% fetal bovine serum (Sigma-Aldrich, St. Louis, MO, USA), 2 mM glutamine (Life Technologies, Carlsbad, CA, USA) and 10 mM HEPES (Life Technologies). The MC38 cell line (MC38-CEA; Kerafast, Boston, MA, USA) was cultured in high glucose DMEM supplemented with 10% fetal bovine serum, 2 mM glutamine, 5 mM HEPES and 0.1 mM non-essential amino acids (Life Technologies). Both cell lines were cultured at 37 °C with 5% CO_2_, and were routinely tested for mycoplasma.

### Treatment experiments in mice

All procedures and experiments including mice were approved by the National Animal Research Authority in Norway (Application number 11654), and conducted according to the regulations of European Laboratory Animals Science Association^29^ and the ARRIVE guidelines^30^. Female Balb/c and C57Bl/6 mice (age 5–12 weeks, weight 18–24 g) were purchased from Janvier (Le Genest-Saint-Isles, France), and kept in specific pathogen free environment, at constant temperature (22 ± 1 °C) and humidity (62 ± 5%), 15 air changes/hour and a 12 h light/dark cycle. Food and water were supplied ad libitum, and the mice were given paper and cardboard houses for environmental stimulation. MAK III cages were used to house the mice, and maximum 10 mice were kept in each cage and the different treatment groups were mixed in the cages. For treatment experiments, 6–9 mice were included in each group. For histology experiments, 4 mice were included in each group. Animals were sacrificed using cervical dislocation when the s.c. tumors reached 15 mm, or if the animals showed signs of illness or discomfort according to standardized criteria.

Tumors were established by s.c. injection of 2.5 × 10^6^ CT26 cells or 1 × 10^6^ MC38 cells using an injection volume of 100 µL. Peptide treatment was initiated when the tumors reached a mean of ~ 80 mm^3^ (± 20 mm^3^). Tumor size was measured by caliper, and the volume was calculated using the formula: width^2^ × length × 0.5. The peptides DTT-205 and DTT-304 were dissolved in 0.9% NaCl to a final concentration of 10–30 mg/mL and 50 µL solution (0.5–1.5 mg) or vehicle (NaCl) was injected intratumorally for 2–4 consecutive days. The treatment was generally well tolerated, but three out of 91 animals treated died shortly after the first or second injection (two C57Bl/6 mice treated with DTT-304 (1 mg) and one Balb/c mouse treated with DTT-205 (1 mg)). Animals were considered cured if s.c. tumors disappeared and did not regrow within 35 days.

### Rechallenge

For splenic injection of tumor cells, the mice were anesthetized with sevoflurane (Baxter, Deerfield, IL, USA). Buprenorphine (0.15 mg/kg) and carprofen (5 mg/kg) were given s.c. prior to the surgical procedure for postoperative pain relief. The spleen was exteriorized through a transverse laparotomy in the left hypochondrium, and 50 µL of cell suspension containing 0.5 × 10^6^ CT26 cells or 1.0 × 10^6^ MC38 cells, was injected 2–3 mm into the splenic parenchyma. Splenectomy was performed seven minutes after injection of cells after ligating the splenic vessels using titanium clips (Ligaclip; Ethicon, Cincinnati, OH, USA) to avoid tumor growth in the spleen that would affect survival, preventing assessment of growth in the liver. The wound was closed using absorbable sutures (Polysorb 5-0, Covidien, Dublin, Ireland). Five of the C57Bl/6 mice (four previously treated with DTT-205 and one treated with DTT-304) died shortly after splenic injection. At autopsy, each liver containing tumor was excised and weighed before the tumor tissue was dissected and weighed separately. If the mice did not develop tumor within 60 or 80 days, for experiments with CT26 and MC38 respectively, the mice were sacrificed as it was then assumed they would not develop tumor.

### Histology

Tumors were fixed in 4% formaldehyde and paraffin embedded, and sections were stained with hematoxylin and eosin (HE).

### Assessment of adverse effects

Male Sprague–Dawley (Charles River, Portage, MI, USA) rats (320–400 g) were instrumented with DSI (St. Paul, MN, USA) telemetry transmitters. Solutions of DTT-205 and DTT-304 were prepared for i.v. injection at dose volumes of 0.5 mL/kg/dose in 0.9% NaCl. Doses were filtered through a 0.2 μM polypropylene (PP) filter prior to injection. Each rat was manually restrained and vehicle or DTT solution was dosed twice intravenously over approximately 1–2 min. The injections were separated by approximately 240 min. The first dose was 0.3 mg/kg, while for the second injection the dose was increased to 1.0 mg/kg. Thus, from 0 to 240 min the test line represents 0.3 mg/kg, while > 240 min it represents 1.0 mg/kg. Heart rate (HR), mean arterial pressure (MAP), diastolic arterial pressure (DAP), systolic arterial pressure (SAP), pulse pressure (PP) and body temperature (BT) were recorded for − 1 to 24 h following the first dose via telemetry and reported in 10 min averages from − 1 to 24 h following the first dose. This study was performed by CorDynamics Inc. (Chicago, IL, USA).

### Degradation time study

For degradation studies with trypsin and chymotrypsin, the peptides were dissolved in a concentration of approximately 1 mg/mL in a buffer consisting of 50 mM NH_4_HCO_3_ for trypsination and 50 mM Tris–HCl (pH 7.8), 1 mM CaCl_2_ for chymotrypsination. Trypsin or chymotrypsin was added in a 1:100 enzyme/peptide weight ratio and incubated at 37 °C in the UPLC sample manager with injections (5 µL) immediately after addition of enzyme and thereafter every 13 min until all peptide was degraded. If the peptides did not show sign of degradation, the experiments were ended after 52 min. The samples were analyzed on a Waters UPLC H-class with a PDA detector equipped with a Waters Acquity UPLC BEH C18 1.7 µm 2.1 × 50 mm column. Separation was obtained with a gradient starting with 5% solvent B with a linear increase to 50% B over 10 min. The column compartment was set to 60 °C and the sample compartment to 37 °C to mimic body temperature on the sample. One injection was made every 13 min to determine how fast DTT-205 and DTT-304 are degraded by trypsin and chymotrypsin. The trypsination and chymotrypsination experiments were ended after 13 min and 52 min, respectively. The reduction of peak areas of the intact peptide were determined over time using integration software and was expressed as a percentage of the original peak area (i.e. before enzyme was added) and was plotted as a function of time. Each experiment was done in triplicate.

### MRGX2 agonist fluorimetric assay

Evaluation of the agonist activity of DTT-205 at the human MRGX2 receptor (UniProt nr Q96LB1) expressed in rat basophil leukemia cells, determined by measuring their effect on cytosolic Ca^2+^ ion mobilization using a fluorimetric detection method based on Kamohara et al.^31^ was performed by Eurofins-Cerep SA (Celle-Lévescault, France). Chem-1 cells without (null cells) and with (recombinant cells) induced expression of human MRGX2 receptor were suspended in HBSS buffer (Thermo Fisher Scientific, Waltham, MA, USA) complemented with 20 mM HEPES and seeded at a density of 6.5 × 10^4^ cells/well in 96-well plates. The following day, medium was removed and cells were washed with assay buffer (20 mM HEPES, 2.5 mM Probenicid). Loading buffer (100 µL, assay buffer with 5 mM Fluo8 Dye (AAT Bioquest)) was added and incubated for 1.5 h at room temperature. The plate was placed in the microplate reader FLIPR Tetra with a run time of 180 s. The ligand DTT-205 was dissolved in dimethyl sulfoxide (DMSO) and concentrations of 0.1, 1, 10, and 100 µM, 10 µM PAMP-20 (CAS nr. 150238-87-2) or HBSS buffer (basal control) were added to the cells after 10 s. Cells were excited at 470–495 nm and emission was detected at 515–575 nm. The cellular agonist effect, i.e. calcium flux, was calculated as a percentage of the response to the known reference agonist PAMP-20.

### Cancer cell panel screening

The MTS cell viability assay was used to assess the efficacy of DTT-205 in several cancer cell lines. Adherent tumor cells were grown as monolayer and non-adherent tumor cells were grown in suspension at 37 °C in a humidified atmosphere (5% CO_2_). Cells were grown in their respective culture media. For treatment experiments, cells were seeded in 96-well plate and incubated at 37 °C overnight in medium supplemented with FBS before treatment. Before DTT-205 treatment, the cells were washed in serum-free RPMI-1640 and fresh medium (90 μL) without serum was added. DTT-205 was dissolved in RPMI-1640 (without FBS) and a concentration range from 0.02 to 100 µg/mL was prepared (9 points). Cells were incubated in triplicate for 4 h in a 100 µL final volume of culture medium containing DTT-205 at 37 °C with 5% CO_2_. Triton X100 (final concentration of 2% v/v) was used as a positive control. At the end of incubation, 20 μL of a 0.22 μm freshly filtered combined solution of MTS (2 mg/mL) and PMS (0.92 mg/mL) in Dulbecco’s Phosphate Buffered Saline (DPBS) were added in each well. Culture plates were further incubated for 1 to 4 h depending on the cell line at 37 °C. Optical Density (OD) was measured at 490 nm in each well using Envision microplate reader (PerkinElmer, Courtaboeuf, France). This study was performed by OncoDesign (Dijon, France).

### Statistical analyses

Statistical analyses were conducted using GraphPad Prism v7 (GraphPad Software, LaJolla, California, USA) or SPSS 21 (IBM, Armonk, NY, USA). Survival curves (Kaplan–Meier plot) were compared using log-rank test. Differences in tumor weight and calcium flux were calculated using unpaired t-test. Analyses of changes in cardiovascular parameters and body temperature were performed by calculating area under curve (AUC) from the time of injection and in the 60 following minutes and comparing the values using unpaired t-test. p-values < 0.05 were considered statistically significant.

## Supplementary Information

Supplementary Information.
