# Supplementary material for: Oncolytic peptides DTT-205 and DTT-304 induce complete regression and protective immune response in experimental murine colorectal cancer
Source: Sci Rep. 2021 Mar 24;11:6731. doi: 10.1038/s41598-021-86239-6 (PMC7991660; doi:10.1038/s41598-021-86239-6)
Supplement: Supplementary file 1 — Supplementary Information. [file 41598_2021_86239_MOESM1_ESM.docx]

**Oncolytic peptides DTT-205 and DTT-304 induce complete regression and protective immune response in experimental murine colorectal cancer**

**Karianne Giller Fleten^1,2^, J. Johannes Eksteen^3^, Brynjar Mauseth^4^, Ketil André Camilio^4^, Terje Vasskog^5^, Baldur Sveinbjørnsson^4,6^, Øystein Rekdal^4^, Gunhild M. Mælandsmo^1,6,^**^#^**, Kjersti Flatmark^7,1,2,#,^**^*^

**Supplementary figures**

**
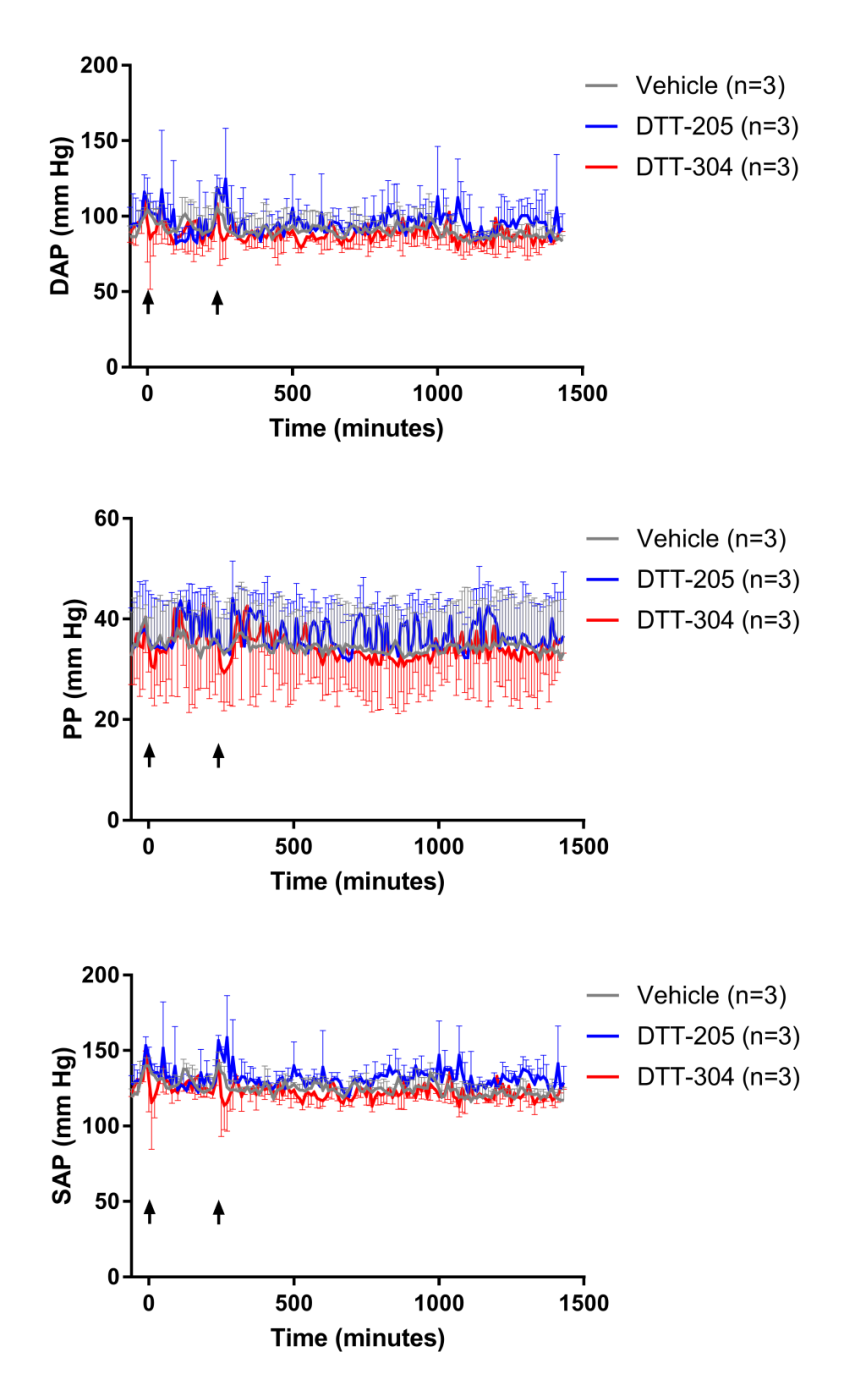
**

**Supplementary Figure 1.** **Cardiovascular assessment in rats after treatment with DTT-205 or DTT-304.** Diastolic arterial pressure (DAP), pulse pressure (PP) and systolic arterial pressure (SAP) pulse in rats treated with DTT-205 or DTT-304. Arrows indicate time of injection. Error bars indicate standard deviation.


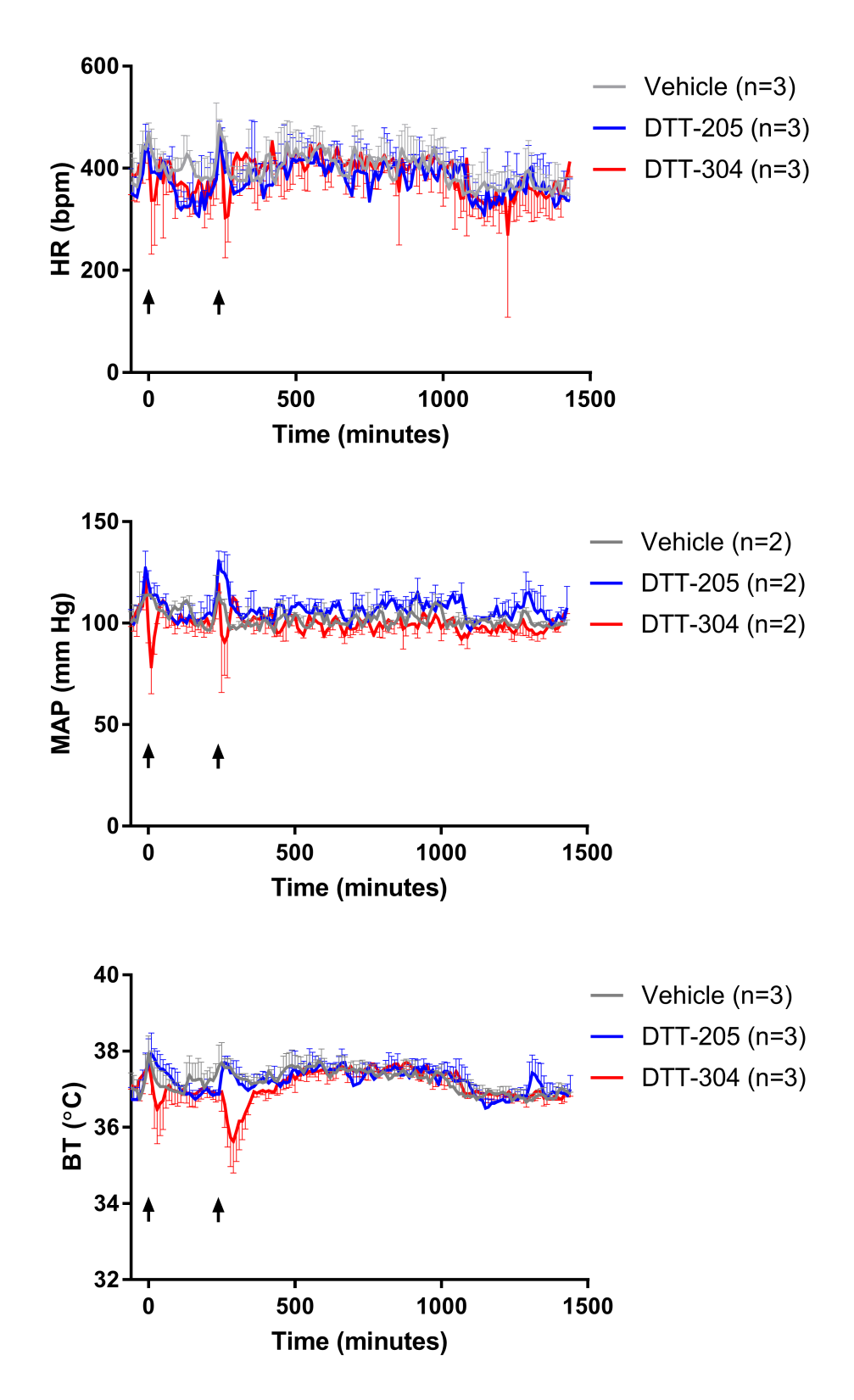


**Supplementary Figure 2.** **Cardiovascular assessment in rats after treatment with DTT-205 or DTT-304.** Heart rate (HR), mean arterial pressure (MAP) and body temperature (BT) in rats treated with DTT-205 or DTT-304. Arrows indicate time of injection. Error bars indicate standard deviation.

**
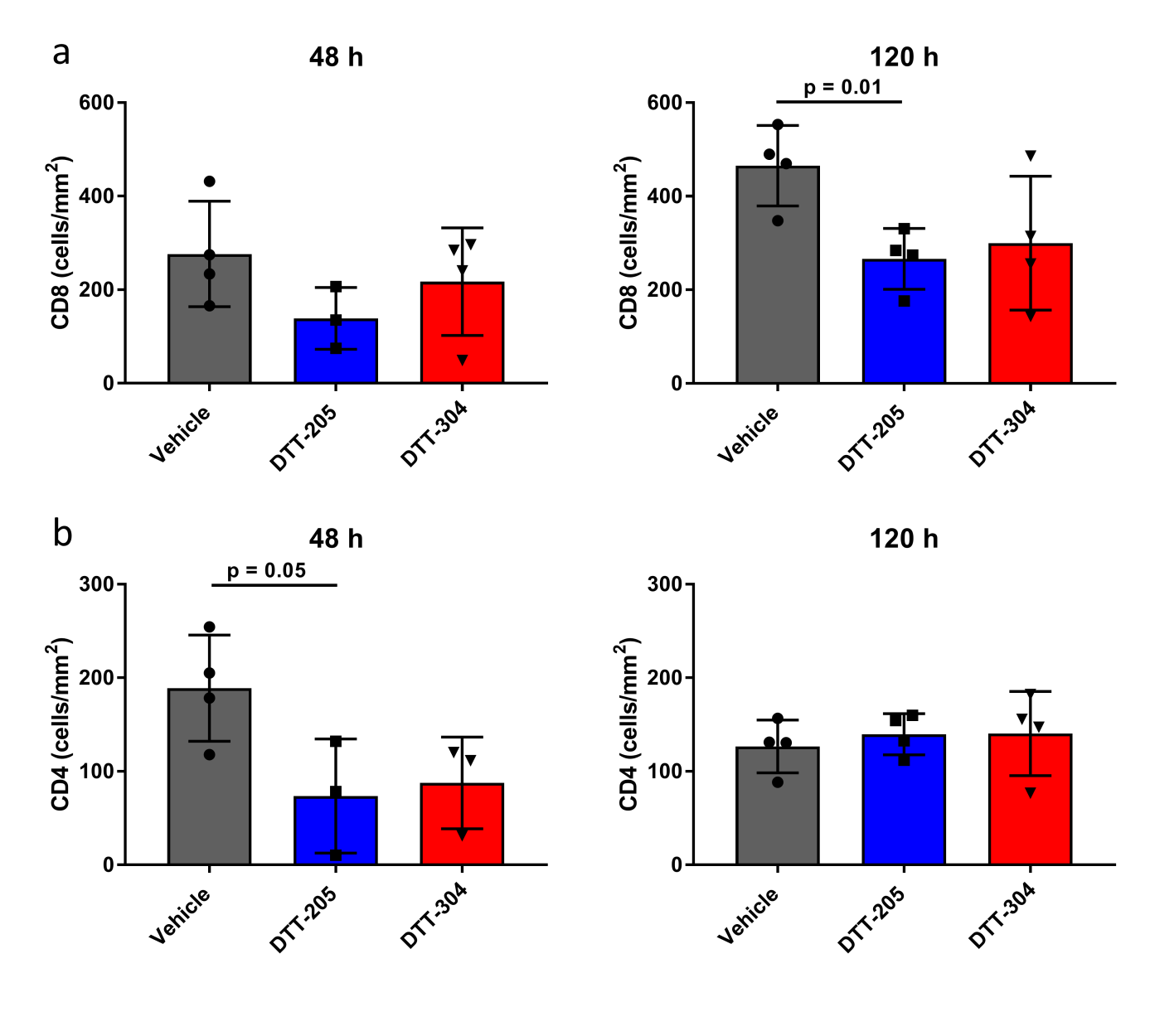
Supplementary Figure 3. T-cell infiltration in MC38 tumors after treatment with DTT-205 or DTT-304.**

Quantification of CD8 (a) and CD4 (b) positive T-cells in MC38 tumors treated with two injections of 1 mg DTT-205, DTT-304 or saline. Tumors were harvested 48 and 120 h after first peptide injection. Error bars indicate standard deviation. Large inter- and intra-tumor heterogeneity were observed. A significant decrease in CD8 cells after 120 h and in CD4 cells after 48 h in tumors treated with DTT-205 were observed.

Immunohistochemical staining was performed using the DAKO EnVision FLEX system (Dako, Glostrup, Denmark). Deparaffinization, rehydration and target retrieval were carried out using a PT-link and FLEX Target Retrieval Solution (high pH). Sections were treated with EnVision Peroxidase-Blocking Reagent for 5 minutes to block endogenous peroxidase, before incubation over night at 4 °C with primary rabbit monoclonal anti-CD4 dilution 1:1000 (clone ab183685, Abcam, Cambridge, UK) or rabbit monoclonal anti-CD8 dilution 1:1000 (clone ab209775, Abcam). The sections were then incubated with Labelled Polymer HRP anti-rabbit for 30 min and stained for 10 min with 3`3-diaminobenzidine tetrahydrochloride (DAB), counterstained with hematoxylin, dehydrated and mounted in Cytoseal XYL. T-cells were manually counted by KGF. T-cell densities were counted in 1-4 regions (area of each region, median 1.33 mm^2^) depending on the size of the tumor. Differences in T-cell infiltration were calculated using unpaired t-test.
